# Predicting factors of medical treatment success with single dose methotrexate in tubal ectopic pregnancy: a retrospective study

**Published:** 2015-06

**Authors:** Fariba Mirbolouk, Azadeh Yousefnezhad, Atefeh Ghanbari

**Affiliations:** 1 *Department of Obstetrics and Gynecology, Guilan University of Medical Sciences, Rasht, Iran.*; 2 *Social Determinants of Health Research Center, Guilan University of Medical Sciences, Rasht, Iran.*

**Keywords:** *Tubal pregnancy*, *β-hCG*, *Methotrexate*

## Abstract

**Background:**

Nowadays, The first step in treatment of ectopic pregnancy (EP) is medical treatment. Medical treatment with methotrexate (MTX) for EP is safe and effective method without the risks associated with the surgical procedure. But there are controversies between studies for which patients will respond better to medical treatment.

**Objective:**

The aim of the present study was to investigate the predictive factors of success or failure of treatment of EP with single dose MTX.

**Materials and Methods:**

In this retrospective study, records of 370 patients who were treated for tubal EP with single dose of MTX were reviewed during four years. Patients were divided into two groups; the first group or “success group” are the patients who were successfully treated with MTX. The second group or “failure group” consist the patients who did not respond to the MTX therapy. The week of gestation, size and location of EP and β-hCG level were compared between groups.

**Results:**

Of 370 patients, 285 (77.1%) were successfully treated with MTX. 85 patients (22.9%) required surgery after a mean of 5.4 (range 2-15) days. Day-1 beta- human chorionic gonadotropin (β-hCG) and fall in β-hCG between day 1 and day 4 were the best predictors for single dose MTX treatment success. The cutoff value of initial β-hCG with the success treatment results was found to be 1375 IU/mL there was no statistical difference between groups about week of gestation, size and location of EP.

**Conclusion:**

The results showed that patients who have β-hCG levels below 1375 and the number of cases with decreasing β-hCG level on day 4 are the good candidates for medical treatment.

## Introduction

An ectopic pregnancy (EP) is one of the major causes of maternal morbidity and mortality. Late diagnosis leads to rupture and cause internal hemorrhage (1). Nowadays, due to scientific, laboratory and imaging technologies advances, EP diagnosed at an early stage with transvaginal ultrasonography and serum Beta-human chorionic gonadotropin (β-hCG) assay (2, 3). Compared to previous treatment, although instead of laparotomy, laparoscopy is preferred, medical treatment with methotrexate (MTX) seems to be more attractive to doctors. Medical management of unruptured EP with intramuscular MTX is common and cost effective (4). Although there is still controversy regarding the appropriate treatment protocol. A meta-analysis estimated the overall success rate of single dose protocol to be 88.1% with a 95% CI: 86-90%. The failure rate of single dose administration of MTX was estimated to be 1.96 times higher than the use of multi dose treatment (5).

Several studies to determine factors associated with the success or failure of response to treatment was done. Women most likely to respond to MTX therapy are thought to be those with small gestational masses, lower serum concentrations of human chorionic gonadotropin and progesterone, and the absence of blood in the peritoneal cavity, but there is controversy in previous studies to determine the true effect of these characteristics on success rates (6). For example, one of the factors associated with successful treatment response is β-hCG level at the beginning of treatment but the value of the determinant or the Cutoff in different studies is varied. One study stated that the failure rate of MTX in β-hCG between 5000-9999 is 13%, 18% between 10000-14999, 32% in human chorionic gonadotropin (hCG) >15000 IU/L and 65% in hCG >4000 IU/L (7, 8).The aim of the present study was to find the predictive factors of success and failure of treatment of unruptured EP with single dose of MTX.

## Materials and methods

In this cross-sectional study, medical records of 370 women admitted with the diagnosis of EP in Alzahra Hospital, the tertiary regional and teaching hospital, Rasht, Guilan during the four year period from October 2009 to December 2013 were reviewed. The study was approved by the Committee for Ethics of Guilan University of Medical Science. The authors of the study were committed to the principles of the Helsinki Convention.

Inclusion criteria were: women with unruptured tubal EP diagnosis, hemodynamically stable, β-hCG titrage under 5000 IU, absent fetal cardiac activity, and who treated with single dose of MTX. Women who treated with double dose protocol of MTX at first, or unstable patients who had laparotomy before medical treatment were excluded. Patients who discontinued medical treatment and left the hospital were excluded, also. Demographic data such as age, marital duration, gravidity, last menstrual period date (LMP), history of abortion, EP, infertility, contraceptive use and clinical presentation such as abdominal pain, vaginal bleeding, and amenorrhea were taken by a check list from patients documents.

Patients received intramuscular MTX at a dose of 50 mg/m^2^ surface area that was calculated from a nomogram with the use of height and actual body weight after written informed consent. A repeat dose of MTX was given if hCG levels did not fall under 15% between days 4 and 7 after dosing or if subsequent weekly hCG levels fell 15%. Successful treatment is considered a 15% drop in hCG between days 4-7 or between days 11-14 after second injection. Surgical intervention took place in cases of tubal rupture and in patients who did not respond to MTX treatment. Tubal rupture diagnosis was on the basis of hemodynamic and clinical signs such as rapid blood pressure drop, increased abdominal pain, and the presence of blood in the abdomen cavity confirmed by ultrasound.Patients were divided into two groups; the first group or “success group” are the patients who were successfully treated with MTX. The second group or “failure group” were the patients who did not respond to the MTX therapy. These women were initially been treated with MTX but underwent surgery after they had shown no positive response to the medical therapy or had a tubal rupture.

**Statistical analysis**

Statistical analysis was done via SPSS software (Statistical Package for the Social Sciences, version 16, SPSS Inc, Chicago, Illinois, USA) Student’s *t* test was used to compare means and chi-square (^2^) or Fisher exact tests were used when appropriate to compare dichotomous variables. Receiver operator characteristics (ROC) curves for initial β-hCG concentration were created to establish cut-off points associated with success in both groups. P˂0.05 was considered statistically significant.

## Results

The Mean age of women was 29.34±5.57 years old (range 17-48). The presenting symptoms were abdominal pain with vaginal bleeding (57.8%), vaginal bleeding (18.1%), abdominal pain (17.8%), and amenorrhea (6.2%). Of 370 patients, 285 (77.1%) were successfully treated with medical treatment with MTX. 85 patients (22.9%) required surgery after a mean of 5.4±4.2 (range 2-15) days. In both the success and failure groups, the age of the patients (mean 29.31 and 29.44 years, respectively), the week of gestation (mean 6.99 and 7.05 weeks, respectively), the size of EP (mean 28.30 and 30.20 mm, respectively), the location of tubal EP (Right or Left), number of gravidity, infertility or EP history, contraceptive use, revealed no statistically significant differences (Table I).

**Figure 1:**
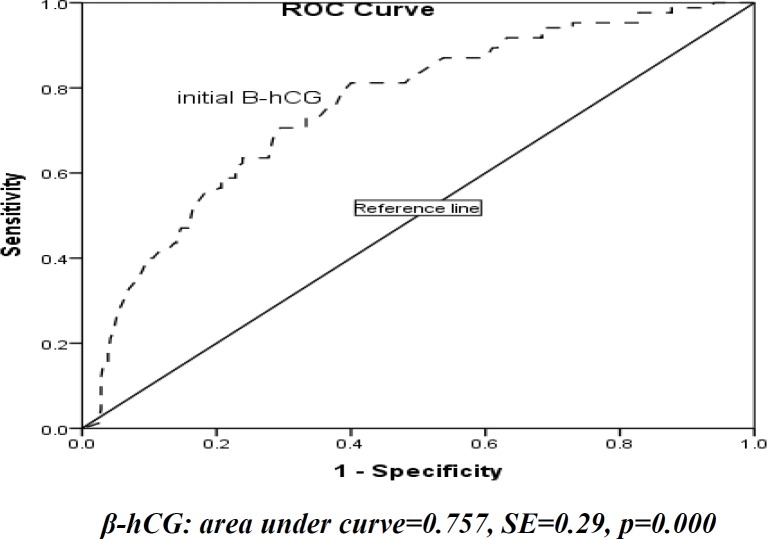
Receiver operating characteristic (ROC) curves for initial β-hCG concentration on successful outcome

There was statistically significant difference between the groups in number of abortion. Frequency of abortion in failure group was higher than success group (0.41 and 0.22 respectively) (p=0.03). The medians of β-hCG levels on days 1, 4, and 7 were significantly higher in the "failure group" (2541 vs. 1167, 2807 vs. 1132, and 2723 vs. 931 mIU/mL, respectively) (p=0.0001). Falling in serum hCG between days 0-4 of treatment in failure and success group were 38.8% and 63.9%, respectively (p=0.0001). 23.5% in success group and 36.5% in failure group required more than one single dose of MTX (p=0.001). With the help of ROC curve analysis, we managed to establish the cutoff point for the β-hCG serum level. At the value of 1375 mIU/mL, sensitivity and specificity for prediction of failure of treatment with MTX reached 70% and 70.5%, respectively (Figure 1). Failure rate among patients with hCG >1375 IU/L was 41.7% vs. 11.1% in patients with a lower level (p=0.01). 48 (13%) patients experienced some side effects. Abdominal pain (6.82%) and GI complication such as vomiting (1.11%), and nausea (5.10%) were the most complication.

**Table I:** Patients and ectopic pregnancy characteristics

**Characteristics**	**Success group ** **n=285 (22.9%)**	**Failure group ** **n=85 (22.9%)**	**p-value **
Age (year)٭	29.31 ± 5.60	29.44 ± 5.50	0.84
Marital duration (year)	5.79 ± 4.42	6.39 ± 4.16	0.82
Live (n)	117 (41.1%)	35 (41.2%)	0.92
Abortion (n)	58 (22.3%)	28 (33%)	0.71
Ectopic pregnancy (n)	21 (7.4%)	6 (7.1%)	0.12
Infertility history (%)	92 (32.3%)	34 (40%)	0.18
Gestational age (week)	6.99 ± 1.63	7.05 ± 1.73	0.81
Size of EP (cm)	28.30± 10.79	30.20 ± 10.77	0.15
Endometrial thickness(mm)	9.07 ± 4.39	9.18 ± 4.69	0.83
hCG 1 day (mean)	1167	2541	0.0001
hCG 4 day (mean)	1132	2807	0.0001
hCG 7 day (mean)	931	2723	0.01
MTX injection (n)	67 (23.5%)	31 (36.5%)	0.001

hCG: human chorionic gonadotropin

MTX: Methotrexate

٭ Continuous data presented as mean ± SD with p-values obtained from Independent-Samples* t*-test; Categorical data presented as n (%) with p-value obtained from Chi-Square test.

## Discussion

MTX therapy for EP of all routes has a success rate of between 74% and 84% (2). Potter *et al* reported a success rate of 85% (69/81 patients), this was 72.4% (63/87) in Ustunyurt *et al*, 89% in Bottin *et al*, 88% in Orozco *et al*, and 75% (30/40) in Vaswani *et al* study (3, 9-12). Success rate of MTX therapy in our study was 77.1% that is the same as the other studies. Success rate in one study in Iran that carried out by Behnamfar *et al* was 78% and 18.7% required second dose that was 23.5% in our study (13). Based on previous studies β-hCG level >5000 and presence of FHR in EP would be reduced the success rate of medical treatment, there for we excluded three patients from our study (14). The β-hCG level on days 1, 4 and 7 in our study were significantly higher in the failure group which was similar to Cohen *et al* study (15). In Potter *et al* study the median pretreatment serum beta-human chorionic gonadotropin level was lower in those women in whom treatment was successful compared with those women with treatment failure (793 vs. 3804 mIU/mL, p<0.002), similar to Ustunyurt *et al* study, (1,417 mIU/mL vs. 5,995 mIU/mL, p<0.001) (9, 10). Based on our findings number of cases with decreasing β-hCG level on day 4 was significantly more in the success group compared to the failure group (38.8% and 63.9%, respectively) similar to Ustunyurt *et al* (61.9 and 37.5%, respectively), Nguyen *et al*, Vaswani *et al*, and Skubisz *et al* (3, 10, 16, 17). At the value of 1375 mIU/mL, sensitivity and specificity for prediction of failure of treatment with MTX reached 70% and 70.5%, respectively. In Markwitz *et al* study at the value of 1790 mIU/mL, sensitivity and specificity in the success group reached 81% and 78%, respectively (18). At the value of 5921 mIU/ml in Vaswani *et al* study sensitivity and specificity were 100% and 93.33% in success group (3). There was no significant difference between groups about age of patients, gravidity, history of EP, infertility, contraceptive use, and size and location of ectopic mass similar to Barnhart *et al* and Lipscomb *et al.* None of these variables would predicted the success of MTX treatment in this population study (14, 19). Logistic regression analysis demonstrated that day-1 and fall in 1-4 days β-hCG level was the significant independent variables for prediction of MTX treatment outcome. The side effects of MTX are related to the dose and mode of administration. The incidence varied from 2% with local injection to 21% in those treated systematically. With single-dose MTX, most authors have reported no significant adverse effects (14). In our study the abdominal pain (6.8%) and GI complication such as vomiting (1.1%), and nausea (5.1%) was the most seen complication.

## Conclusion

In conclusion, we found that with initial serum chorionic gonadotropin concentration we can select good responder patients with ectopic pregnancies to single dose MTX protocol. And patient with β-hCG level under 1375 should expect better results. The weaknesses of the study were the retrospective nature, and incomplete records in some cases. Alzahra Hospital is a referral center in Guilan province and there is diversity in its patients, there for results of our study could be generalized to society. However, prospective studies with larger sample size, considering ethnic differences are necessary.

## Note

***This article extracted from residential thesis. (Azadeh Yousefnezhad)***
